# Inhibitory Control in Bilinguals and Musicians: Event Related Potential (ERP) Evidence for Experience-Specific Effects

**DOI:** 10.1371/journal.pone.0094169

**Published:** 2014-04-17

**Authors:** Sylvain Moreno, Zofia Wodniecka, William Tays, Claude Alain, Ellen Bialystok

**Affiliations:** 1 Rotman Research Institute, Baycrest Centre for Geriatric Care, Toronto, Ontario, Canada; 2 Department of Psychology, University of Toronto, Toronto, Ontario, Canada; 3 Department of Experimental Psychology, Jagiellonian University, Krakow, Poland; 4 Department of Psychology, York University, Toronto, Ontario, Canada; University of Rome, Italy

## Abstract

Bilinguals and musicians exhibit behavioral advantages on tasks with high demands on executive functioning, particularly inhibitory control, but the brain mechanisms supporting these differences are unclear. Of key interest is whether these forms of experience influence cognition through similar or distinct information processing mechanisms. Here, we recorded event-related potentials (ERPs) in three groups – bilinguals, musicians, and controls – who completed a visual go-nogo task that involved the withholding of key presses to rare targets. Participants in each group achieved similar accuracy rates and responses times but the analysis of cortical responses revealed significant differences in ERP waveforms. Success in withholding a prepotent response was associated with enhanced stimulus-locked N2 and P3 wave amplitude relative to go trials. For nogo trials, there were altered timing-specific ERP differences and graded amplitude differences observed in the neural responses across groups. Specifically, musicians showed an enhanced early P2 response accompanied by reduced N2 amplitude whereas bilinguals showed increased N2 amplitude coupled with an increased late positivity wave relative to controls. These findings demonstrate that bilingualism and music training have differential effects on the brain networks supporting executive control over behavior.

## Introduction

Conscious self-regulation of thought and action is mediated by executive functions (EF), a system that includes sub-components such as goal planning, self-monitoring, decision making, attention, mental flexibility, and inhibition [1], [2]. These supervisory functions are vital to regulating other cognitive processes [3] and are important predictors of development and life outcomes [4]. Research over the past several decades has established a robust inherited component to EF (e.g., [5]), but it is also clear that environmental factors in the form of specific experiences can give rise to significant individual differences. Two common training experiences, bilingual language use [6] and its development [7] and the acquisition of music skills [8], have both shown robust effects on EF development in children even though the training does not specifically target EF or its components. Both training experiences have also demonstrated effects on aging: musical training acts as a buffer from the deleterious effects of age-related sensory loss [9] and bilingualism has been shown to protect against cognitive decline in aging (e.g., [10], [11]). However, the common outcome of these experiences on executive functions is not clear, specifically regarding the timing and nature of the neural correlates of the behavioral effects. In this study, we investigate whether these experiences influence cognition and behavior through similar or distinct mechanisms. The purpose is to determine how experience modifies brain function by comparing two different experiences and assessing whether they have different influences on performance in a simple inhibition task. The results will contribute to our understanding of the mechanisms involved in experience-related plasticity. Previous studies have shown better EF performance in nonverbal tasks by both bilingual children ([12], [13], see [14] for meta-analysis) and adults ([15]–[18]; see [19] for meta-analysis) compared to their monolingual counterparts. The tasks typically involve conflict between the correct response and a misleading alternative, as in the flanker task [20], Simon task [10] or Stroop task [15].

The conflict created by the jointly activated languages in bilinguals was investigated in a study by Rodriguez-Fornells and colleagues [21] using scalp recordings of event-related potentials (ERPs). They adapted a standard go-nogo task to a picture naming paradigm by asking participants to name only the pictures that met a criterion (e.g., name begins with a vowel) and refrain from naming pictures that did not (e.g., name begins with a consonant) and found that bilinguals were able to efficiently suppress the processing of words in the non-target language. In a follow-up study, Rodriguez-Fornells et al. [22] compared monolinguals and bilinguals in a go-nogo naming task and found that conflict stimuli in which the name of the picture in the two languages was associated with a different response evoked a fronto-central negativity for the bilinguals. Moreover, monolinguals showed the expected N2 effect associated with nogo trials, but for bilinguals the N2 was delayed by about 200 ms and showed a larger amplitude than that found for the monolinguals. These results were interpreted as evidence of the involvement of EF, particularly inhibition, in bilingual language processing. They may also reflect a stronger suppression of the competing response plan, although the delay in latency could also be due to the possibility that response competition evokes an extended computation period. No studies to date, however, have compared ERP waveforms for monolingual and bilingual adults on a standard nonverbal go-nogo task and connected these findings to the previous research on nonverbal EF tasks.

The proposed mechanism for the bilingual processing advantages in executive control is that bilingual language use necessarily recruits the EF system to manage attention to two jointly activated (and potentially conflicting) languages. This experience in which EF abilities are constantly employed during language processing results in a more efficient inhibitory control system for bilinguals than for monolinguals (see [23] for a review), presumably because of the massive practice and reorganization of that system during language selection. Support for this interpretation comes from fMRI evidence showing that the same networks are involved in both nonverbal executive control and language switching in bilinguals [24], leading to information processing advantages in the brain regions responsible for nonverbal executive control in bilinguals [25].

Like bilingualism, music training has also demonstrated an influence on EF [26]–[28]. Miyake and Shah [29] noted that music training involves working memory, selective attention and inhibition, task switching, updating and monitoring − all components of EF [1]. For example, George and Coch [30] used ERPs to investigate the relationship between music training and working memory using standardized tests of working memory, standard auditory and visual oddball paradigms. Their findings showed a relation between musical experience and higher WM performance in both auditory and visual modalities.

This impact of music training on EF has also been reported for children after only 4 weeks of exposure. Moreno et al. [8] compared the benefits of a music training program for 5-year-old children to an equally engaging visual arts training program. In one task, children performed a nonverbal go-nogo paradigm while EEG was recorded. After training, children in the music training group were better able to discriminate go from nogo trials than were children in the visual arts training group. Moreover, the music lessons induced a functional brain change wherein children showed early differentiation of go from nogo trials in their P2 response (a positive deflection of the ERP peaking at about 200 ms after stimulus), whereas no changes were seen in the later N2/P3 complex. Previous research has shown the P2 to be sensitive to stimulus categorization [31], such that increased amplitude reflects enhanced processing of relevant stimulus features. More specifically, in the go-nogo task, the P2 response has been observed in go trials and interpreted to reflect the activation of stimulus-response pairings [32]. Thus, either music training influences relatively early stages of information processing or the brief training offered in this study was insufficient to induce broader functional brain change in later information processing stages. These possibilities need to be disentangled by studying musicians with more substantial experience.

Although both bilingualism and music training have an impact on EF, their effects are not identical. Bialystok and DePape [26] compared the effect of musical training and bilingualism on conflict processing in three groups: bilinguals with no musical training, monolingual musicians, and monolinguals with no musical training. The conflict tasks were a nonverbal Simon task based on position-direction conflict and an auditory Stroop task based on pitch-word conflict (the word “high” sung in a low note). All participants performed equivalently on background cognitive measures and the control conditions for the two EF tasks, but performance diverged in the conflict conditions. In the Simon task, both bilinguals and musicians outperformed monolingual controls, but in the auditory Stroop task, the musicians outperformed participants in the other two groups. The interpretation was that bilingualism and music training have some common benefit to EF as shown by conflict resolution in the spatial Simon task, but that music training additionally imparts unique benefits, at least for tasks requiring attention to auditory stimuli. The study focused strictly on behavior; However, it may be the case that a similar brain network is influenced by both musical training and bilingualism: Moreno and colleagues showed more bilateral scalp potentials in EF tasks for both bilinguals [33] and musicians [8] than was found for their respective comparison groups, but the two trained groups were not directly compared.

Documenting the similarities and differences in the way that these two experiences influence inhibitory control has broad implications for understanding the mechanisms associated with experience-induced plasticity. Current interest in cognitive reserve [34] and brain training programs for older adults [35] indicates broad acceptance that such plasticity is possible and my produce effective interventions for cognitive decline, yet there is little understanding about how these changes actually occur. Comparing the neural responses of musicians and bilinguals, groups that have been shown to produce behavioral improvements in inhibitory control, is a crucial first step in understanding these neural mechanisms.

In the present study we use a nonverbal go-nogo task to compare how bilinguals and musicians resolve conflict created by infrequent nogo relative to go trials. In the go-nogo task, the ERP signatures observed when participants must withhold a prepotent response on nogo trials are increased amplitudes in N2 and P3 waves, relative to go trials [36]. Although the functional role of the N2 and P3 remains a matter of debate (e.g. [37]–[39]), both components appear to reflect top-down responses to prepotent response tendencies. This view is supported by data showing that complexity increases N2 and P3 latencies. However, functional differences are apparent between the components as increasing difficulty reduces the amplitude in the P3 response but not N2 (e.g., [32] for a recent test of the effect of task complexity) and that covert/imagined responses (e.g. when a participant imagines the correct response while making no actual movement) have intact N2 but reduced P3 [40]. Manipulating the parameters of the go-nogo task has lead to the view that the nogo N2 reflects either the conscious registration of response conflict [41], [42] or inhibition of the prepotent motor plans [43], whereas the nogo P3 response is associated with overt inhibition of a response or with the monitoring of the outcome of the intention to inhibit (e.g., [44]).

If the bilinguals' and musicians' executive functions advantage is caused by similar mechanisms of neuroplasticity, ERP responses in both groups should be similar to each other but different than the control group. The benefits of both music training and bilingualism are often linked with detection of competing response alternatives and inhibitory control over behavior, as discussed above. Therefore, we take the view that greater inhibition of prepotent response plans will result in larger amplitude N2 responses in both expert groups relative to controls. However, different mechanisms of neuroplasticity in musicians and bilinguals may differ in other components associated with the go-nogo task. In accordance with research on the music training on the P2 [8], [45] we hypothesize that musicians will present an altered early P2 response reflecting an advantage in representing stimulus response associations relative to controls [32]. In contrast, bilinguals are expected to exhibit altered late P3 responses [22], [33] reflecting an extended monitoring of the appropriateness of the selected response [44]. Overall, our expectations are that the individual experiences created by music training and bilingualism have dissociable mechanisms for influencing control over behavior.

## Methods

### Ethics statement

The study was approved by the Baycrest Research Ethics Board, and the rights and privacy of the participants were observed. Each individual provided written informed consent in accordance with the guidelines established by the University of Toronto and Baycrest Centre for Geriatric Care. Participants received monetary compensation for their time.

### Participants

Eighteen English monolingual, 14 English monolingual musicians, and 18 bilingual non-musician volunteers between the ages of 18 and 33 years old participated in the study. All participants had normal or corrected-to-normal vision. Data from four participants were discarded due to excessive ocular artifacts; data from three other volunteers were discarded as a result of high alpha rhythm occurrence. The final sample was composed of 15 monolingual English speakers, 13 English speaking monolingual musicians, and 15 bilingual non-musician adults. Demographic information is presented in Table 1. The lower number of English monolingual musicians is explained by the difficulty in finding musicians who speak only one language with the same education background as comparison groups. Over 40 additional musicians were screened but not included due to their language background.

**Table 1:** Background information for participants with variable ranges in brackets.

Group	N	Gender	Handedness	Mean Age	Mean Years of Education
Control	15	4 M, 11 F	15 R	23.6 (19–27)	16.4 (13–21)
Musician	13	4 M, 9 F	1 L, 12 R	26.5 (21–38)	18.0 (15–21)
Bilingual	15	15 F	1 L, 14 R	23.0 (18–32)	16.9 (13–23)

Monolingual participants (both controls and musicians) were all born and raised in either Canada or the United States. Bilingual participants were born in Canada (5), Russia (1), Romania (1) or Israel (8) and in addition to English spoke Hebrew (9), Russian (1), Romanian (1) and French (4). Eight bilinguals had some knowledge of a third language (average self-rated proficiency on 0–100 continuum was 43.4). Bilingual participants not born in Canada immigrated during childhood (ages ranged from 1 to 15 years), except one participant who immigrated at 30 years old but reported having learned English at 8 years of age. Thirteen of the fifteen bilingual participants learned L2 before age 12 (*M* = 6.2 years) and two participants were late learners: one at 15-years old and one at 14-years old (final group *M* = 7.2). Only one bilingual reported having English as his first language, yet six bilinguals considered English as their dominant language. Ratings for proficiency in the dominant language were higher (*M* = 97.1) than those for the non-dominant language (*M* = 81.9). English-speaking musicians (n = 13; 9 female) were amateur instrumentalists with an average of over 12 years of private or group lessons of continuous training in Western classical music on their principal instrument (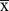 = 12.1, sd = 6.2 years). The majority of musician participants played multiple instruments (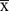 = 2.7, sd = 1.2 instruments) whereas the remaining three played one instrument. Piano (i.e., 10 participants) was the primary instrument for most musicians. English-speaking nonmusicians (n = 15; 11 female) had no more than 5 years of formal music training on any combination of instruments throughout their lifetime, nor had they received formal instruction within the past 5 years. Most of the nonmusicians (i.e., 24) reported not having followed any music training at all or playing an instrument.

All participants completed a test of receptive vocabulary knowledge in English, and the 12 bilinguals for whom Hebrew or French was the other language also completed this test in their second language (Standard PPVT English score:103; Hebrew score:109; French score:124). All participants filled out a language background questionnaire. On average, bilinguals reported speaking English 26% of the time at home and 85% at work, and reported hearing English 38% of the time at home and 89% at work.

### Procedure

#### Psychometric Testing

The psychological assessment battery included the Language and Social Background Questionnaire, Peabody Picture Vocabulary Test (PPVT III) for receptive vocabulary, Cattell Culture Fair Intelligence [46] for fluid intelligence, and Corsi Block test for spatial working memory. The purpose was to establish the comparability of participants across groups on these measures.

#### Go-nogo ERP Paradigm

Participants were seated in a comfortable chair in an acoustically and electrically shielded room. Geometrical shapes were presented on a computer monitor 50 cm from the participant. A chin-rest was used to fix the distance of presentation, align the participant's line of sight with the center of the screen, and reduce head movement artifacts. There were 4 different stimuli created from two types of shapes (triangles or squares) in two different colors (white or purple) to reduce stimulus repetition effects. Each trial consisted of the following events: a colored shape was presented on a black background for 186 ms followed by a variable blank screen interstimulus interval lasting 1500, 2000, or 2500 milliseconds to prevent strong expectancy effects. Participants were instructed to press a key on a standard keyboard in response to white shapes as quickly and accurately as possible (75% probability) and to withhold responding to purple shapes (25% probability). The experiment lasted 20 minutes and consisted of 576 trials (432 go and 144 nogo trials). A practice block of 20 trials was used to familiarize participants with the task. Stimuli were displayed using E-Prime version 1.2 (Psychology Software Tools, Inc.). The order of trials was randomized across participants. During the task, participants did not receive any feedback on their performance.

#### ERP recording and analysis

Electrophysiological activity was recorded continuously from an array of 64 electrodes with a bandpass of 0.05–100 Hz and sampling rate of 500 Hz using NeuroScan Synamps2 (Compumedics, El Paso, TX, USA). Electrodes were referenced to Cz during recordings and re-referenced to an average reference for data analysis. Electrodes were placed at the superior/inferior orbital rim and outer canthi to monitor vertical and horizontal eye movements.

ERPs were created for correct trials only using Brain Electrical Source Analysis (BESA, V.5.1.8) software. The analysis epoch included 200 ms of pre-stimulus activity and 1000 ms of post-stimulus activity. Artifact detection was carried out in two stages. First an automatic rejection amplitude threshold of 100 µV was used to exclude prominent artifacts. These data were then manually scanned to exclude remaining artifacts with amplitude approaching +/−75 µV. These thresholds allowed at least 90% of correct trials to be retained for each participant.

For each participant, a set of ocular movements was obtained prior to and after the experiment [47]. From this set, averaged eye movements were calculated for both lateral and vertical eye movements as well as for eye-blinks. A principal component analysis of these averaged recordings provided a set of factors that best explained the eye movements. The scalp projections of these components were then subtracted from the experimental ERPs to minimize ocular contamination such as blinks, saccades, and lateral eye movements for each individual average. ERPs were then digitally low-pass filtered to attenuate frequencies above 20 Hz (zero phase; 24 dB/oct).

Mean and peak ERP amplitudes were measured in selected latency windows based on prior research and are listed for each respective component in the results section. Mixed model ANOVAs were computed using SPSS on amplitude data with group as a between-subjects factor and condition (go, nogo) and electrode as within-subject factors. When ANOVA analyses violated the homogeneity of variance assumption, the Huyn-Feldt adjustment was used to gauge significance levels (uncorrected degrees of freedom are presented). Post-hoc tests relied on the Neumen-Keuls procedure where appropriate. Analyses were originally done with midline and lateral electrodes electrodes (FC1/2/3/4, C1/2/3/4, CP1/2/3/4, PO3/4/7/8) as factors in ANOVAs. However, no laterality effects reached significance (all *p*s>.10) so analyses using midline electrodes only are presented for simplicity. Our focus was on group differences in the nogo condition, so follow-up analyses for significant group by condition interactions used one-way ANOVAs calculated separately on go and nogo conditions. Data will be made freely available upon request.

## Results

### Age and Education

Age and years of education were analyzed with one-way ANOVAs for group. There was a marginally significant effect of age, *F*(2, 42) = 3.1, *p* = .051, showing that the musicians group was older (26.5 years) than the bilingual (23.5 years) and the controls (22.9 years). Years of education were not significantly different between groups (*p*>.4). Correlations between demographic measures (age and education) and task measures (behavior and ERPs) did not reveal any predictive relationship for any group (all *r*-values <.35).

### Psychometric Testing

PPVT, Corsi, and Cattell standardized scores were analyzed with one-way ANOVAs for group. No significant group effect was found for PPVT or Cattell scores (all *p*s>.16). There was a significant effect of group on Corsi spatial working memory score, *F*(2, 42) = 3.3, *p*<.05, *η_p_^2^* = .14, revealing an advantage in visual-spatial memory for musicians relative to controls (*p*<.05) but no significant difference between bilinguals and either controls or musicians (all *p*s>.20).

### Go-nogo Behavioral Data

Behavioral data for the go-nogo task are presented in Table 2. Response time data for correct go trials were analyzed with a one-way ANOVA for group and revealed no significant difference, *F*<1. Accuracy data were analyzed using a *d*-prime score computed for each participant and again revealed no differences between groups, *F*(2,40) = 1.27, *p* = .29.

**Table 2:** Mean percentage accuracy (and standard error) and response time (and standard error) for go and nogo trials.

Group	Go Correct (%)	Go RT (ms)	Nogo Correct (%)	D′
Control	95.8 (1.5)	329 (9.7)	92.3 (1.3)	3.81 (.25)
Musician	96.8 (1.4)	340 (10.1)	92.4 (1.9)	3.94 (.26)
Bilingual	91.3 (2.8)	332 (12.1)	95.6 (1.4)	3.67 (.36)

### ERP Responses

**Figure 1:**
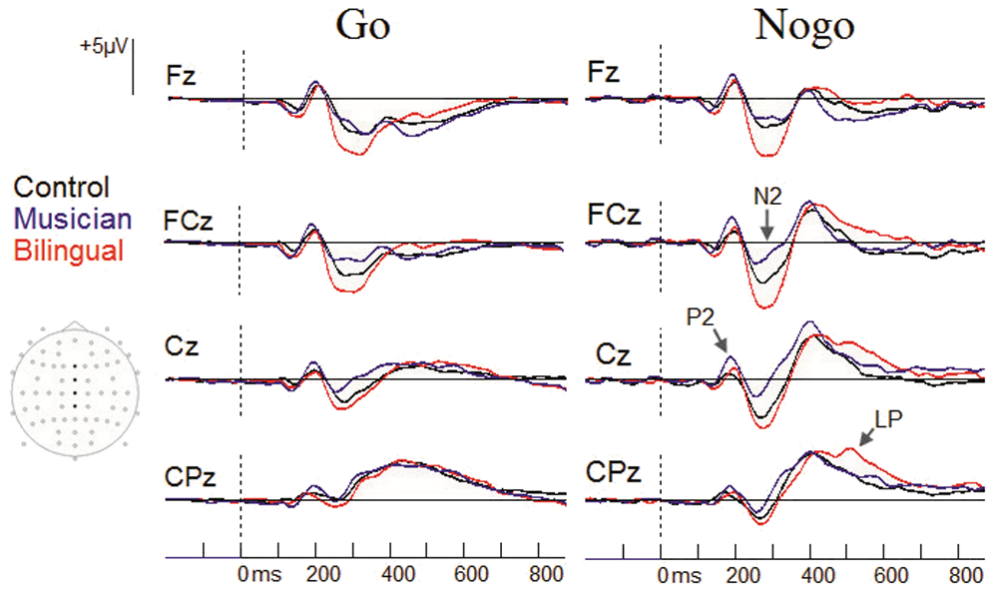
Grand average waveforms from go and nogo trials for bilinguals, musicians, and controls; representing the differences between groups on P2, N2 and LP waveforms at Fz, Cz, Pz and CPz.

**Figure 2:**
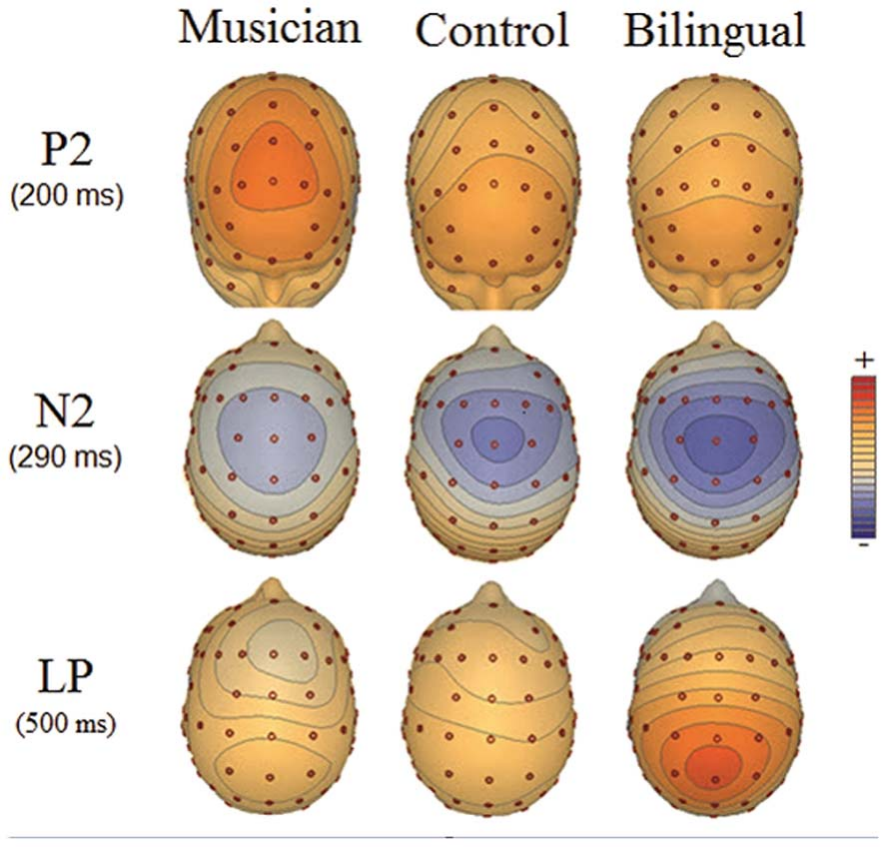
Topographic maps of ERP waveforms (P2, N2 and LP) from the nogo condition across musicians, controls and bilinguals. Each gradient represents a change of approximately 0.5 µV.

Visual inspection of ERP waveforms revealed the expected N2 and P3 waves. Although P2 effects are not typically associated with the nogo task, previous research has highlighted their relevance for identifying the effects of music on cognitive functioning [8]. The three groups did not differ in mean amplitude or condition effects in early visual evoked responses (i.e., P1 & N170) between 70–170 ms after stimulus onset (all *p*>.10). However, group differences appeared at about 200 ms after stimulus onset at fronto-central sites (i.e., P2 wave) as well as at about 300 ms in the N2 wave at central sites. A central-parietal P3 effect was observed across groups (≈425 ms), but this was followed by a protracted positive wave in some participants (≈525 ms) that we refer to here as the late positivity (LP) effect. ERP overlays showing go and nogo responses are presented in Figure 1, while topographic maps of relevant components are presented in Figure 2.

#### P2 Results

Across individual participants, P2 waveforms had short waveform durations and a modestly wide variation in latency making peak scoring an effective method for detecting condition differences. Given the frontal-central distribution of the P2 deflection, peaks and accompanying latencies were scored at Fz, FCz, and Cz in the time interval 180–230 ms. A condition (go, nogo) by electrode (Fz, FCz, Cz) by group (bilingual, musician, control) ANOVA on peak amplitude revealed an effect of electrode, *F*(2,80) = 3.80, *p*<.05, *η_p_^2^* = .15, in which amplitude decreased from anterior to posterior sites. There was also a trend towards a group effect, *F*(2,40) = 2.73, *p* = .06, *η_p_^2^* = .15, in which the musician group produced larger P2 waves than either bilinguals or controls. There was no effect of condition or any interaction effects (all *p*s>.25). Mean ERP values for each wave are shown in Table 3. A similar analysis of P2 latencies was conducted, but there were no main effects or interactions (all *p*s>.30).

**Table 3:** Mean peak amplitude (P2) and average amplitudes (N2, P3, LP) for each group.

	P2 (Cz)		N2 (FCz)		P3 (Cz)		LP (CPz)	
Group	Go	Nogo	Go	Nogo	Go	Nogo	Go	Nogo
Control	.96	.74	−2.68	−3.28	0.71	3.22	1.01	1.51
	(.38)	(.50)	(.54)	(.51)	(.66)	(.81)	(.57)	(.79)
Musician	2.13	2.63	−1.28	−1.35	1.35	4.20	1.34	1.90
	(.34)	(.56)	(.84)	(.88)	(.95)	(1.40)	(.72)	(1.02)
Bilingual	1.12	1.54	−4.21	−5.49	0.80	3.35	1.48	3.09
	(.35)	(.29)	(.94)	(.97)	(.65)	(.74)	(.60)	(.60)

Standard errors are shown in brackets.

#### N2 Results

The N2 response and subsequent slow-wave ERPs showed similar latencies across participants and were analyzed based on mean amplitude measures. Mean amplitudes were calculated for the N2 at 270–320 ms at electrodes Fz, FCz, and Cz. A condition by electrode by group ANOVA revealed a main effect of condition, *F* (1,40) = 8.32, *p*<.01, *η_p_^2^* = .29, a main effect of electrode, *F* (1,40) = 7.97, *p*<.01, *η_p_^2^* = .17, and a main effect of group, *F* (2,40) = 4.23, *p*<.05, *η_p_^2^* = .18, as well as a three-way interaction of these factors, *F* (4, 80) = 8.32, *p*<.01, *η_p_^2^* = .29. The topographic distribution of N2 waves was more slightly more anterior in musicians than in controls or bilinguals. All three groups showed robust N2 responses at FCz. To simplify the analysis of group and condition, average amplitudes at this site were used in a simple effect analysis. The condition by group ANOVA at FCz revealed effects of condition, *F* (1,40) = 7.68, *p*<.01, *η_p_^2^* = .16, and group, *F* (2,40) = 5.88, *p*<.01, *η_p_^2^* = .22, and an interaction between them, *F* (2,40) = 3.6, *p*<.05, *η_p_^2^* = .16, superseded main effects. Simple effects analyses showed no group difference for go trials, *F*(2,40) = 2.41, *p*  = .11, but a significant group difference for nogo trials, *F* (2,40) = 5.54, *p*<.01, *η_p_^2^* = .22. Pairwise comparisons revealed that the bilinguals had larger N2 responses than musicians (*p*<.01) and controls (*p*<.05) and that musicians showed significantly smaller N2 responses than controls (*p*<.05). The condition by group effect on N2 amplitude could also be followed up by testing the contrast of go vs nogo in each group at FCz. This repeated measures analysis reveals significant N2 effects in monolingual controls, *t*(14) = 6.09, *p*<.01, and bilinguals, *t*(14) = 5.32, *p*<.01 but not musicians, *t*(12) = 1.22, *p* = .24. Additionally, contrasting the N2 difference waves showed larger N2 effects in bilinguals than controls, t(28) = 2.05, p<.05, and a marginal reduction in N2 difference wave in musicians compared to controls, *t*(26) = 1.81, *p* = .08.

#### P3 & LP Results

All three groups produced the expected larger P3 wave in nogo relative to go trials, but there were minimal differences in this effect between groups. The component was measured as the average amplitude between 350–500 ms across electrodes FCz, Cz, and CPz. The group by condition by electrode ANOVA revealed a condition by electrode interaction, *F*(2,80) = 12.4, *p*<.01, *η_p_^2^* = .21. This was explained by a larger P3 effect (go vs. nogo) at the central and frontal-central channels that rapidly decreased at the posterior electrodes. A simple effects analysis of go vs. nogo trials at the peak electrode (i.e., Cz) revealed a robust effect of condition, *F*(1,40) = 51.40, *p*<.001, *η_p_^2^* = .56. The overall P3 amplitude did not differ between the groups (*p* = .76) or show an interaction with group (*p* = .93).

An LP effect immediately followed the P3 and was seen as a continuation of the difference between go and nogo trials, particularly in the bilingual participants, and had a more posterior extension to its topography than the P3. Mean amplitudes were measured in the interval between 475–575 ms at FCz, Cz, and CPz electrodes. The condition by group by electrode ANOVA showed no main effects, all *F*s<2, but did reveal a trend towards a group by condition interaction, *F*(2,40) = 2.16, *p* = .09, *η_p_^2^* = .11. This tentative effect was followed up by an exploratory simple effect analysis of group for each of the go and nogo conditions at CPz, where the LP was largest. The group differences in LP amplitude was not significant for go trials, *F*<1, but groups did differ for nogo trials, *F*(1,40) = 3.08, *p*<.05, *η_p_^2^* = .14. Pairwise comparisons showed that bilinguals had larger amplitudes than controls (*p*<.05) and musicians (*p* = .07), who did not differ from each other (*p* = .72).

## Discussion

Three groups of participants with comparable background measures performed a go-nogo task and, in spite of achieving equivalent behavioral performance, produced significantly different neural responses. Specifically, bilinguals produced larger amplitudes for the relevant N2 waveforms on nogo trials than the other groups, and musicians produced smaller amplitudes. These results indicate that the neural plasticity that follows from bilingualism and musical training takes a somewhat different course with differences that are not evident in the behavioral data alone.

Current behavioral results showed no differences between groups. When using simple tasks that most participants can complete with little difficulty, it is not uncommon for groups with known differences in information processing to have equivalent behavior but dramatic differences in brain response (e.g., difference in younger and older adults on simple working memory performance [48]. We deliberately chose a simplified task design in order to isolate the underlying neural processes involved in the musicians' and bilinguals' responses to interference relative to controls. With the simplicity of the task, each group approached a ceiling effect with accuracy rates greater than 90% in both go and nogo conditions. This very high performance level reflects the simplicity of the task and leaves little room to observe behavioral differences. We believe that it remains a strong strategy to use simple task designs in exploratory work since these simple tasks facilitate the breakdown and observation of involved neural processes. Evidence for behavioral differences between controls and bilinguals (see [23]) and musicians (see [49]) is compelling, and the focus of the current study was to better understand the unique neural response of expert groups relative to controls.

In the present ERP data, the earliest differential activation between groups in the present study was in the P2 response, in which participants with music experience showed larger amplitudes than controls or bilinguals in both go and nogo trials. The P2 effect is considered to reflect the strength of the neural representation in primates [50] and the ability to preferentially process relevant visual stimulus features in humans [31]. In the go-nogo task, the P2 may additionally reflect the activation of stimulus response pairings [32]. Thus, this component indexes an aspect of the ability to construct a representation of the current task context and the associated behavioral response in the early stages of processing. Previous research in our laboratory has also shown increased P2 amplitude in the go-nogo task in children following 4 weeks of musical training [8]. Importantly, those results revealed a link between increased P2 amplitude and an improvement in verbal processing scores. It may be, therefore, that this early aspect of processing has links to higher cognitive function by facilitating stronger internal representations of behaviorally relevant stimuli. More efficient early representations of information reflected by this early positivity may help explain recent findings of positive associations between P2 amplitude and high-level processes such as memory [51], [52], semantic processing [53], and intelligence [54]. In the present study, the larger amplitude P2 waveform in musicians may reflect a specific benefit of this expert group in earlier processing and representation of stimuli and the appropriate response (or non-response) pairing. This efficiency in appropriate response pairing then reduces the need for cognitive control processes indexed by later components, such as the typical N2 and P3 [37], [38], [41], [42].

The most striking difference between groups was found in the N2 response − a component suggested to reflect either the detection of conflict between competing response plans [41] or the selective inhibition of the prepotent response plan [40]. There was a graded amplitude between groups in which the N2 effect was minimal in the musician group, modest in controls, and maximal in bilinguals. Thus, even though bilinguals and musicians produced equivalent behavioral performance on this task and have previously shown similar EF benefits (e.g., [26]), the underlying cortical response for these two groups is different. The information processing mechanisms influenced by each experience are dissociable and perhaps lead to differential development or reorganizations of the supporting neural networks. Expertise with a musical instrument has been shown to be related to robust bidirectional activation in auditory sensory and motor areas that create specialized sensory-motor networks [55]. These areas are plausibly involved in musical performance and, therefore, form a unique pathway for musicians to perform such tasks as the go-nogo paradigm used in the present study. Therefore, musical expertise may enable more efficient dissociations of desired and undesired stimulus-response planning at the level of the P2, and subsequent levels of conflict or demands for inhibition at the stage of the N2 are reduced. For bilinguals, fMRI studies have shown greater connectivity in frontal regions [56] and functional reorganization of the executive control system [57]. Moreover, associated frontal regions are observed to be active when switching between two jointly activated languages [24], although the engagement of top-down control may occur later in bilinguals than controls [22]. Following the perspective that the N2 reflects a conflict detection signal or inhibition of the prepotent response, the larger amplitude produce by bilinguals would suggest that they are more sensitive in detecting existing response competition or allocating resources to resolve conflict than controls. In sum, both bilingualism and musical training can be seen to differentially influence brain networks responding to conflict.

All three groups showed the expected increased P3 in response to nogo trials relative to go trials [58], but the response was more protracted for the bilinguals than for the other two groups. To distinguish this effect from the typical P3 response, we refer to it as the LP. The isolation of this ongoing cortical potential in bilinguals is consistent with the view that bilingual language use requires increased attention to monitor the demands imposed by two jointly activated languages that constantly provide a source of interference [59]. Pervasive monitoring of linguistic information processing may influence executive control processes in other contexts that require ongoing monitoring or response inhibition, such as the go-nogo paradigm. As the nogo P3 may reflect the closure of the inhibition of the overt response [32] or the ongoing evaluation of the intention to inhibit [44], it may be that bilinguals have a more robust supervisory mechanism to ensure that the desired response outcome was achieved relative to monolinguals. Hence, brain plasticity effects of bilingualism bias the cognitive control network towards more extensive monitoring of interference and this effect can be seen as more sustained activation in comparison to monolinguals. It has been recently reported that a specific form of bilingual training experienced by simultaneous interpreters is related to a greater sensitivity to a mismatch between the meanings of two words within and across the native and non-native languages, as reflected in enhanced N400 [60].

The overall pattern in these results is that the musicians show the greatest differences from the other groups in the early components of performance that are associated with activating appropriate stimulus-response representations and bilinguals show the greatest differences from the other groups in the later components associated with behavior regulation after the activation of these competing behaviors. Specifically, music training modified the P2 and N2 waves and bilingualism modified the N2 and P3 waves. These patterns can be traced to effects of the experience on functional brain organization; musicians obtain extensive practice in visual, auditory, and motor responses and bilinguals obtain extensive practice in inhibiting activation of the non-target language. Thus, with better representation of the stimuli signaling go and nogo trials, musicians perhaps experience a lesser degree of conflict and require less subsequent inhibitory control to perform the task relative to non-musicians. In contrast, bilinguals are more sensitive than monolinguals at detecting interference and applying inhibitory control after entering a state of conflict. In both cases, executive control is effective in carrying out the appropriate response, but the manner in which the correct response is achieved is different for each group.

Previous research has documented the benefits of music training and bilingualism on a variety of EF tasks as well as showing that each group, when individually compared to controls, show unique patterns of brain activation. The present ERP data are the first evidence, to our knowledge, that directly compare cortical responses of musicians and bilinguals and reveal the diverging pathways by which these groups recruit information processing mechanisms in response to behavioral conflict. These results contribute to the larger enterprise of understanding and defining one of the central mechanisms of the human capacity for adaptation.
